# Should We Consider Cancers as Embryonic Diseases or as Consequences of Stem-Cell Deregulation?

**DOI:** 10.4137/cmo.s603

**Published:** 2008-04-29

**Authors:** Jérémy Bastid, Alain Puisieux, Stéphane Ansieau

**Affiliations:** 1 Inserm U590, Lyon, F-69008, France; 2 Centre Léon Bérard, Lyon, F-69008, France; 3 Université de Lyon, Lyon, F-69003, France; 4 Université Lyon 1, ISPB, Lyon, F-69003, France

**Keywords:** cancer, cancer-initiating cells, embryonic genes

## Abstract

Cancers have long been described as the result of successive selections of somatic cells progressively acquiring growth and survival advantages. Such a model was hardly compatible with the obvious heterogeneity of the cancer cell population present in tumors. This heterogeneity rather suggests that mutations hint multipotent cells that, in addition to the resulting proliferation and survival advantages, display differentiation capabilities. Adult stem cells or progenitors display similar properties, supporting the concept that cancers actually originate from these cells. The recent observation that differentiated cells can dedifferentiate and acquire stemness properties suggests an alternative and additional explanation for the origin of “cancer-initiating” cells and reopens the debate of the contribution of somatic cells to cancer progression.

Adult stem cells are normally located in a niche where they remain quiescent until triggered to regenerate a wounded tissue. In physiological conditions, complex protein networks regulate their homing and their proliferation, protect them from apoptosis during migration and control their differentiation in harmony with the wounded tissue. According to the stem cell theory, signals mimicking tissue damages, promoting stem cell recruitment or favoring their proliferation and migration are susceptible to favor tumor progression. Most of the proteins that regulate stem cell migration in the embryo have now been defined as essential for adult stem cell proliferation and survival. Their expression in cancers can therefore been interpreted as a consequence of the stem/progenitor cell origin of cancers. Nevertheless, an increasing number of genes, that fulfill diverse embryonic functions, also turn out to be reactivated in cancers. Interestingly, the deregulation of several of these genes, including *Twist*, *Shp2*, *K-Ras* and *NF-1*, is also associated with developmental diseases such as Saerthre-Chotzen, Noonan and LEOPARD syndromes, strengthening the general idea that cancer could be a developmental disease^1^,^2^. Whether the detection of embryonic proteins is a consequence of the progenitor cell origin of cancers or rather portrays cancers as an embryonic disease is still a matter of discussion. Elucidating this point will require to determine whether embryonic genes are expressed in the original targeted cell or induced at specific and more advanced stages of tumor progression.

To debate this question, we proposed to focus on regulators of the epithelio-mesenchymal transition (EMT). EMT has emerged as a central process during embryonic development. It is involved in the morphogenics processes underlying parietal endoderm formation and gastrulation, as well as during the formation of several organs and tissues, such as the neural crest (NC), the heart, the musculoskeletal system, the craniofacial structures and the peripheral nervous system. Various signaling pathways, including the EGF, HGF, Met, Wnt-β-catenin, TGFβ, Notch and Hedgehog pathways are known to regulate EMT.^3^,^4^ They all converge to a restricted number of transcription factors including the Id and Twist proteins, the homeobox SIP1 as well as the zinc finger proteins Snai1 and Snai2.^5^,^6^ Whereas Id proteins inhibit EMT^7^, Snai1, Snai2, SIP1 and Twist conversely promote the transition.^8^,^9^ All four EMT inducers are essential for mesoderm patterning during embryonic development and are associated with the metastatic potential of cancer cells.^10^–^12^ *In vivo* detection of Snai1 at the invasive front of colorectal cancers, in association with p16^INK4A^ expression, emphasizes the role of the protein in EMT and metastatic potential determination, by promoting growth-arrest and thereby cell synchronization as required for EMT induction.^13^,^14^ How and at what stage of tumor progression are these genes induced? We recently showed that Twist expression overrides oncogene-induced senescence and apoptosis suggesting that it might promote benign to malignant conversion.^15^ Although to be shown, through their anti-apoptotic properties, Snai1 and Snai2 might also be linked to failsafe program escape. In contradiction with a possible role in the earliest steps of tumor progression, Twist and Snai1 expression is induced by hypoxia and known to promote angiogenesis.^16^–^18^ From these observations, one could assume that the reactivation of embryonic genes in premalignant tissues helps cells to override oncogene-induced failsafe programs whereas in hypoxic conditions it favors neo-angiogenesis or cell dissemination to less hostile environments, two possibilities that might not be mutually exclusive. Indeed, the protein expression levels required for each of the functions might differ. There is little data on Twist, Snai1 and Snai2 expression at the earliest steps of tumor progression. Twist has been described as undetectable in premalignant tissues although a weak or a restricted expression to a limited number of cells remain difficult to exclude.^19^–^21^

Although still partial, convergent information tends to indicate that these proteins might also play a role in adult stem cells or progenitors cells. EMT has been associated with the earliest step of stem cell differentiation. Accordingly, Snai1 and Snai2 have been shown to contribute to NC delamination and migration in chicks and Xenopus, although these functions might not be conserved in rodents.^22^–^25^ Additionally, phenotypic examination of *Snai2* deficient mice highlights the role of the protein in the migration of three stem cell populations, melanoblasts, hematopoietic progenitors and germ cells resulting in pigmentation anomalies, gonadal defect and macrocytic anemia.^26^ Snai2 was also found expressed in neurogenin3-positive endocrine progenitor cells, partially differentiated cells derived from the undifferentiated multipotent epithelial cells of the pancreatic duct-like epithelium.^27^ Snai1 was recently described as implied in bone mesenchymal stem cell migration.^28^ Although Snai1, Snai2 and Twist were found being expressed in neural crest-derived corneal precursors,^29^ poor information regarding the status of Twist in progenitor cells is yet available. Altogether, these observations raise the possibility that these transcription factors assume essential functions in stem/progenitor cells by regulating their survival during migration. If we consider that these cells are at the origin of cancers, their detection in tumors is logically expected. Accidental oncogene activation promotes stem/progenitor cell growth. Twist, and potentially Snai1 and Snai2, override oncogene-induced failsafe programs, extend cell growth capabilities and cooperate with oncogenes to promote an EMT, as observed in epithelial cells. Depending on the differentiation status of the progenitor cell affected and the pattern of proteins expressed, the resulting cancer type might vary. As an example, Pax proteins protect precursor cells from apoptosis during their migration through the embryo. Normally undetectable in adult tissues, Pax genes are overexpressed in various cancers, in link with their embryonic role.^30^,^31^ For instance, Pax3, which is important for NC- differentiation into melanocyte during development,^32^ is overexpressed in melanoma.^30^,^33^,^34^ Pax2 and Pax8, which play an important role in early urogenital development, are essential for tumor progression and survival in several cancers of urogenital origins.^31^

Although there is increasing evidence in support of the stem cell theory, does it definitively exclude that initial events might also hint differentiated cells? Various observations clearly indicate that cell differentiation is not an irreversible cellular state. Experimental grafting of human basal cell-depleted epidermal sheets to immunodeficient mice has clearly demonstrated that the dedifferentiation of differentiated epidermal cells into stems cells or stem cell-like cells is part of *in vivo* tissue regeneration.^35^ Similarly renal proximal tubular cell (RPTC) dedifferentiation is thought to be a prerequisite for regenerative proliferation and migration after renal injury.^36^,^37^ Whether this process opens a window sufficient to accumulate the number of mutations required to fully transform the cells is difficult to estimate. However, primary mutations or epigenetic alterations might generate a genetic instability, thus favoring the accumulation of further events. Indeed, recent studies have shown that abnormal epigenetic changes occur at various stages of tumor progression, mainly during the earliest steps of the neoplastic process. For example, the Wnt pathway is known to be abnormally activated in colon cancers through epigenetic changes;^38^ it is reasonable to assume that additional EMT inducers and embryonic pathways are similarly turned on. Whether reactivation of embryonic genes contributes to enhance, or takes direct part in this dedifferentiation program also remains to be determined. Anyway, these observations clearly open an alternative to the stem cell theory for the generation of pluripotent cancer cells. Of note, the recently established link between EMT and stemness acquisition supports such a possibility.^39^ Further investigations are required to fully validate one or both of these cancer-origin theories. In particular, the development of animal models in which stem cells would be marked and followed independently of their differentiation status to unravel their contribution to carcinogen- or mutagen-induced tumors would be of particular help. Elucidating the contribution of embryonic genes to the earliest phases of tumor progression will also be crucial for clarifying the debate around the origin of cancers.
